# Preparation and Properties of Medium-Density Fiberboards Bonded with Vanillin Crosslinked Chitosan

**DOI:** 10.3390/polym15112509

**Published:** 2023-05-30

**Authors:** Yanwei Cao, Chen Qin, Zhengbo Zhao, Zhe Wang, Chunde Jin

**Affiliations:** 1 College of Chemistry and Materials Engineering, Zhejiang A&F University, Hangzhou 311300, China; 2 Key Laboratory of Wood Science and Technology, Hangzhou 311300, China

**Keywords:** MDF, chitosan, vanillin, mechanical strength, adhesive

## Abstract

An eco-friendly medium-density fiberboard (MDF) was prepared using vanillin (V) crosslinked chitosan (CS) adhesive through a hot-pressing process. The cross-linking mechanism and the effect of different proportions of added chitosan/vanillin on the mechanical properties and dimensional stability of MDF were investigated. The results showed that vanillin and chitosan are crosslinked to form a three-dimensional network structure due to the Schiff base reaction between the aldehyde group of vanillin and the amino group of chitosan. At the same time, when the mass ratio between vanillin/chitosan was 2:1, MDF obtained the best mechanical properties, the maximum modulus of rupture (MOR) of 20.64 MPa, the mean modulus of elasticity (MOE) of 3005 MPa, the mean internal bonding (IB) of 0.86 MPa, and the mean thickness swelling (TS) of 14.7%. Therefore, the MDF bonded with V-crosslinked CS can be a promising candidate for environmentally-friendly wood-based panels.

## 1. Introduction

Fiberboard, due to efficient utilization and saving of wood resources in the forest industry, has been widely used in the world. Wood-based fiberboard adhesives have made major progress because wood bonding technology depends on wood adhesives [1]. Among them, urea-formaldehyde adhesive, phenolic adhesive, and melamine- formaldehyde adhesive were representative. However, the release of free formaldehyde in the fiberboard products produced by these products exceeds the standard significantly [2]. As people realized that free formaldehyde was harmful for health, and facing the depletion of fossil fuels, chemical-based adhesives were changing to reflect the sustainable development of bio-based adhesives.

In the 19th and 20th centuries, it was made from natural resources such as collagen, fish, starch, and other derivatives. Among them, sustainable regeneration of natural biomass adhesives, such as tannic acid, protein, polysaccharide, and lignin, has attracted people’s attention [3]. However, they are generally not used alone. As wood derivatives, they have mechanical properties, poor water resistance, modification, and complex problems related to preparation [4]. Therefore, it is necessary to develop a simple chemical crosslinking modification to convert renewable biomass energy into wood adhesives with high performance, dimensional stability and no formaldehyde for MDF [5].

Chitin is the second most abundant polysaccharide found in nature directly after cellulose. Chitosan is a derivative from chitin and only a part of chitin is transformed in chitosan [6]. Since it has good adhesion and mechanical properties as well as biocompatibility and biodegradability, it has been widely used in many fields, for example wood, biology, medicine and so on [7,8,9]. A large number of highly active amino and hydroxyl groups exist on the straight chain structure to provide bonding between materials, and can react with a variety of chemical groups for chemical modification to synthesize a better effect of biomass adhesive [10]. In recent years, not only has the application of chitosan-based adhesives in the wood field has helped wood materials obtain good mechanical properties, but also the whole biological adhesive based on chitosan, combined with other substances, has been developed successfully [11,12]. However, the poor mechanical properties of the chitosan linear structure limit the expansion of its application. In order to overcome this defect, a stable three-dimensional network structure is formed by chemical crosslinking [13,14]. JI et al. [15] prepared a lignosulfonate/chitosan-based adhesive by making use of glutaraldehyde as a crosslinking agent. Through the mechanical property test, results showed that the MDF prepared with the mass ratio of glutaraldehyde/chitosan between 0.25~0.75 met China’s MDF national standard. Huang et al. [14] utilized 2, 5-dimethoxy-2, 5-dihydrofuran-crosslinked CS as an environmentally-friendly binder to prepare environmentally-friendly medium-density fiberboard through the hot-pressing method. The results indicated that crosslinked chitosan could effectively improve the mechanical properties and dimensional stability of wood-based panels. Xi et al., prepared three-layer laboratory plywood to test the adhesion property of environmentally-friendly chitosan adhesive prepared by specific oxidation modification with glucose, sucrose and starch as hardener [16]. The results suggested that oxidized carbohydrate could effectively improve the bond strength and water resistance of chitosan adhesive, and has good bonding performance. However, the low toxicity of crosslinking agent is a threat to health. This is a new attempt to use the natural crosslinking agent, crosslinking chitosan, as an adhesive for medium-density fiberboard.

Vanillin (4-hydroxy-3-methoxybenzaldehyde) is available both naturally and synthetically. Natural vanillin is abundant in the structure of the pods of naturally occurring vanilla seeds. Compared with it, synthetic vanillin, using chemical synthesis or eugenol and ferulic acid of renewable resources as natural raw materials, has greater advantages in terms of cost [17]. It is often used as a food flavor additive or an intermediate in cosmetics and pharmaceuticals, due to its low cost, environmental protection, and biological functionality. At the same time, it belongs to the phenolic group in structure and represents an ideal nontoxic crosslinking agent for chitosan.

In this paper, the MDF with good physical and mechanical properties using vanillin-chemically-crosslinked chitosan as adhesive was successfully prepared through the hot-pressing process. The cross-linking mechanism of the adhesive and the effect of different proportions of vanillin-cross-linking chitosan on medium-density fiberboard were studied. It provides ideas and reference significance for the preparation of environmentally-friendly fiberboard.

## 2. Materials and Methods

### 2.1. Materials

Mixed wood fiber (F), moisture content 13~15%, Zhejiang New Wood Material Technology Co., LTD. (Zhejiang, China). Chitosan, 90% deacetylation, Shanghai Yuanye Biotechnology Co., LTD. (Shanghai, China). Acetic acid, analytically pure, supplied by Lingfeng Chemical Co., LTD. (Shanghai, China). Vanillin, analytical pure, molecular weight value (152.149), Shanghai Yuanye Biotechnology Co., LTD. (Shanghai, China).

### 2.2. Preparation of Crosslinked Chitosan Binder (CS-V)

In this study, the methods of preparing crosslinked chitosan adhesive are the same, however the difference lies in the change of the V experimental dose as shown in Table 1. For instance, the mass ratio of V to CS was 0.5:1. An amount of 3.5 g of acetic was added into 270 mL of deionized water under magnetic stirring to form an aqueous solution of acetic acid (1.3% *w*/*v*). CS (3.24 g) was added to the acetic acid/water solution, meanwhile stirred in a bath of 70 °C with a magnetic stirrer at 200 rpm for 10 min, so that the chitosan was completely dissolved into the acetic acid solution to form 1.2% chitosan solution. Then, V (1.62 g) was added into the chitosan solution, and the magnetic stirrer was used at the rotational speed of 200 rpm for 30 min. The solution was fully stirred, in order to dissolve V completely into the chitosan solution. The prepared vanillin-crosslinked chitosan solution was placed at room temperature for cooling and standing for 6 h defoaming.

### 2.3. Preparation of Wood-Based Fiberboard Bonded by V-Crosslinked CS (F-CS-V)

In this study, wood-based fiberboard was prepared in the same way. For example, the mass ratio of vanillin to chitosan was 0.5:1 prepared MDF. First, wood fiber (20 g) was dried in the oven at 103 °C after wood fiber was removed of impurities, so that moisture content was measured in an absolutely dry state at 13%~15%. Secondly, chitosan adhesive was evenly sprayed on the surface of the wood fiber (83 g absolute dry mass) by spraying and stirring for several times, so that the chitosan adhesive was fully mixed with the wood fiber. The mixed wood fiber was stirred in a 10-mesh screen to mix well and evenly. The plate vulcanizer was preheated and the temperature risen to 220 °C, kept stable for 1 h. Oil-based baking paper (30 cm × 30 cm) and 100 mesh stainless steel mesh (30 cm × 30 cm) were laid on the steel plate (35 cm × 35 cm). The crosslinked chitosan/wood fibers were then hand-paved multiple times in a mold (20 cm × 20 cm). The billet was prepressed after paving was completed. The fiber slab was placed between stainless steel mesh, oil-based baking paper and steel plate. At the same time, the slab was hot pressed using a thickness gauge of 3 mm to control the thickness of the fiberboard. The target fiberboard (200 × 200 × 3 mm) was packaged in a heat-resistant sealed bag and loaded at room temperature for 12 h to prevent bending and deformation. A table saw was used to cut off 2 cm of the edge to produce a 160 × 160 × 3 mm plate with target density of 0.8 ± 0.02 g cm^−3^. By the same method, the fiberboard prepared without adhesive was named F-W, the fiberboard prepared with V was named F-V, and the fiberboard prepared with CS was named F-CS.

### 2.4. Physical and Mechanical Properties Test

According to China’s Medium-Density Fiberboard national standard (GB/T 11718-2021) test method, the static bending strength and elastic modulus of the specimen (150 mm × 50 mm × 3 mm) were tested by a universal mechanical testing machine (5960 Instron, Norwood, MA, USA) in a three-point bending mode. The loading rate was 5 mm min^−1^. Specimens (50 mm × 50 mm × 3 mm) were tested for 24 h water absorption thickness expansion rate, in 8 groups. For the internal binding strength test (50 mm × 50 mm × 3 mm), loading rate value (1.0 mm min^−1^), 8 groups were tested.

### 2.5. Characterization

FT-IR was used to test the chemical structure changes of crosslinked chitosan and its mixing with wood fibers after the hot-pressed process (FTIR, IR-Prestige21, Shimazu, Japan). The sample to be tested and the KBr powder were mixed evenly at the ratio of 1:100 and pressed into slices for scanning. The wavelength range was 4000~400 cm^−1^ and the resolution was 4 cm^−1^. SEM was used to observe the internal microstructure of the fiberboard before and after crosslinked chitosan was added (SEM, TM3030, Hitachi, Tokyo, Japan). The samples to be observed were treated with gold spray to avoid damage to the fiber surface structure during the test, The scanning voltage was 15 kV. NMR identifies the skeleton structure of CS, V and CS-V according to the absorption peak intensity and location of chemical shift of the substance to deduces the connection mode between the molecules of the substance. Methanol-d4 was utilized as solvent for ^1^HNMR of vanillin and chitosan derivatives. Additionally, 2% Acetic acid-d4/water solution was used as solvent for CS test ^1^HNMR (Brooke 400M, Biuerica, MA, USA). XRD was used to observe the change of crystallinity before and after vanillin crosslinked chitosan, and the effect of change V:CS (fixed amount) on crystallinity (Shimadzu XRD-6000, Kyoto, Japan). The scanning range was 5~60°, and the scanning rate was 3° min^−1^. TG was used to test the thermal stability of CS before and after the addition of V, as well as the thermal stability of the fiberboard before and after the addition of the crosslinked chitosan adhesive (TG, NETZSCH TG 209 F1 Libra, Sebul, Germany). The temperature range was 30~800 °C, and the heating rate was 10 °C min^−1^. N_2_ was used in the gas atmosphere during the test. XPS was used to analyze the surface elements of fiberboard before and after adding crosslinked chitosan binder (TG, NETZSCH TG 209 F1 Libra, Germany). The test conditions were as follows: excitation source was set as Al Kα ray. Spot size was set as 400 μm. Working voltage of the XPS instrument was set as 12 kV. Filament current was set as 6 mA. The full spectrum scanning energy is set at 150 eV and the step size is 1 eV. The narrow-spectrum scanning energy is set to 50 eV and the step size is 0.1 eV.

## 3. Results and Discussion

### 3.1. Mechanical Properties Analysis

**Figure 1:**
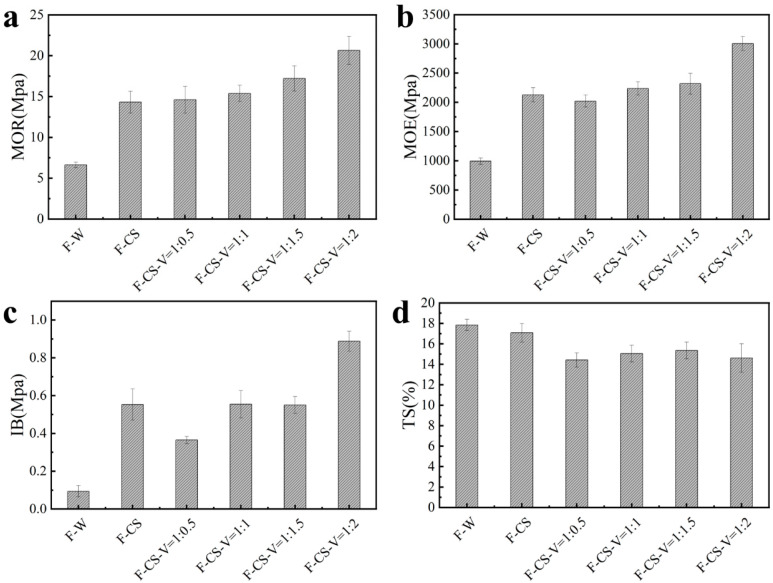
MOR (**a**), MOE (**b**), IB (**c**), TS (**d**) of crosslinked chitosan fiberboard in different proportions and F-W.

Figure 1 shows the effects of different proportions of added crosslinked chitosan binder and F-W on the physical and mechanical properties of MDF. According to Figure 1a to Figure 1c, when the amount of fixed CS was added, MOR, MOE and IB of MDF showed an increasing trend as the ratio of vanillin to chitosan increased. However, in Figure 1d, the swelling rate of water absorption thickness of fiberboard decreased significantly after V was added. In conclusion, when the mass ratio between chitosan and vanillin is 1:2, the physical and mechanical properties of F-CS-V are relatively good, showing a mean value of MOR (20.64 Mpa), the mean value of MOE (3005 Mpa), the mean value of IB (0.86 Mpa) and the mean value of TS (14.7%).

### 3.2. SEM Analysis

**Figure 2:**
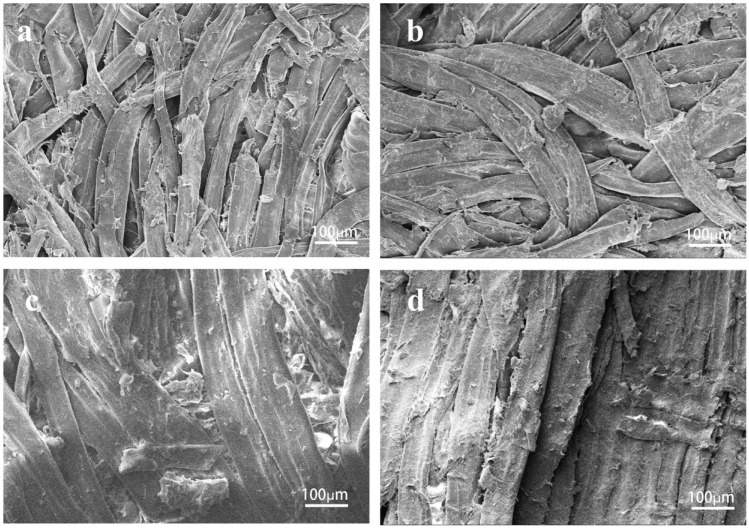
Surface morphologies of F-W (**a**), F-V (**b**), F-CS (**c**) and F-CS-V (**d**).

Figure 2 shows the surface morphologies of different fiberboards F-W (a), F-V (b), F-CS (c), F-CS-V (d). As can be seen from Figure 2a,b, the surface of fibers was smooth and binding between the fibers was loose structure. There is a large pore between fibers result in the weak mechanical properties of fiberboard. In Figure 2c, the fiber surface of F-CS attached adhesive, fiber surface was rough and bonding between fibers was closer. As can be seen from Figure 2d, the fiber surface was clustered by binder wrapped with crosslinked chitosan, therefore fibers were closely bonded, which is the reason for the best physical and mechanical properties.

### 3.3. FT-IR Analysis

**Figure 3:**
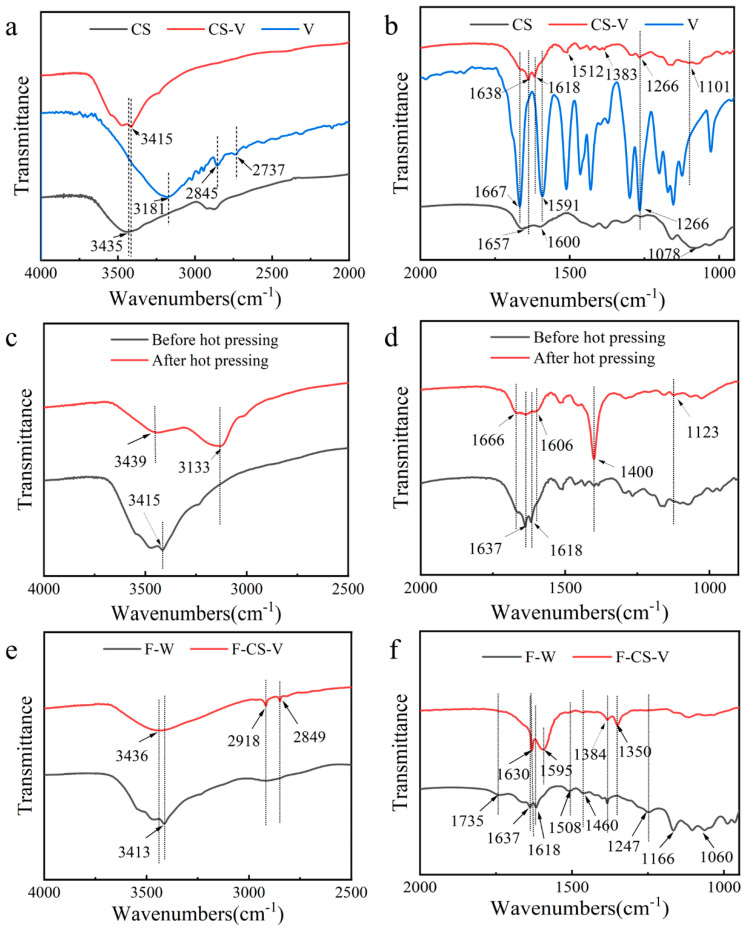
(**a**) and (**b**) are the infrared spectra of CS, V and CS-V; (**c**) and (**d**) are the infrared spectra of CS-V before and after hot pressing; (**e**,**f**) are the infrared spectra of F−W and F-CS-V.

Figure 3 shows the Fourier transform infrared spectra of CS, V, CS-V, F-W, F-CS-V and CS-V after hot pressing. In Figure 3a, 3435 cm^−1^ is the hydroxyl absorption peak of CS. In the infrared curve of V, 3181 cm^−1^ is the hydroxyl absorption peak. The C–H stretching vibration absorption peak of –CH_3_ is 2845 cm^−1^. The C–H stretching vibration absorption peak of –CHO is 2737 cm^−1^. The hydroxyl absorption peak of CS-V is 3415 cm^−1^. In Figure 3b, 1657 cm^−1^ amide (Ⅰ) and 1600 cm^−1^ amide (Ⅱ) are both characteristic peaks of CS. The absorption peak at 1087 cm^−1^ is the C–O stretching vibration absorption peak of chitosan CH_2_-OH [18,19]. In the infrared curve of V, 1667 cm^−1^ is the stretching vibration absorption peak of C=O in –CHO, 1591 cm^−1^ is the vibration absorption peaks of the benzene ring characteristic of V. The bending vibration absorption peak of V phenol hydroxyl group is 1266 cm^−1^ [20]. In the infrared curve of vanillin crosslinked chitosan, the appearance of new peaks of 1618, 1512 and 1266 cm^−1^ were the result of V benzene ring and hydroxyl group, indicating the cross-linking between vanillin and chitosan. The C=N stretching vibration of 1638 cm^−1^ indicates that the amino group of CS reacts with the aldehyde group of V in Schiff base. The C–O stretching vibration absorption peak of CS (CH_2_-OH) moved to 1101 cm^−1^, indicating that a C–O–C structure might be formed, and acetalization reaction occurred between vanillin and chitosan [20,21]. The hydroxyl group of CS was transferred from 3435 cm^−1^ to 3415 cm^−1^, and its strength decreased significantly after crosslinking, which may be due to the hydrogen bond interaction between chitosan and vanillin [22,23,24]. Fourier infrared spectra showed that chitosan and vanillin crosslinked successfully through Schiff base reaction and hydrogen bonding.

Figure 3c shows the infrared spectrum of CS-V before and after hot pressing. The absorption peak of the hydroxyl group before CS-V hot pressing is 3415 cm^−1^. The absorption peak of the hydroxyl group is 3439 cm^−1^, while 3133 cm^−1^ is the absorption peak of V in the infrared curve, after CS-V hot pressing. In Figure 3d, the infrared curve after the hot pressing of CS-V, 1666 cm^−1^ is the C=O stretching vibration absorption peak of V. The absorption peak intensity at 1637 cm^−1^ decreased significantly, which may be due to the inhibition of the Schiff base reaction between vanillin and chitosan under the hot-pressing condition. The characteristic peak of benzene ring at 1618 cm^−1^ moved to 1606 cm^−1^. The absorption peak at 1400 cm^−1^ may be due to the high temperature decomposition of V generation new substances. However, the O–C–O absorption peak was found at 1123 cm^−1^, and the intensity of the absorption peak was significantly enhanced, indicate that the hot-pressing condition was conducive to the acetal reaction of vanillin and chitosan.

In Figure 3e, the maximum absorption peak of hydroxyl group of F-W is at 3413 cm^−1^, and that of F-CS-V is at 3436 cm^−1^, indicating that CS-V and wood fiber are bonded by hydrogen bonds. The new peaks of 2918 cm^−1^ and 2849 cm^−1^ showed that both chitosan and vanillin were loaded on wood fibers. In Figure 3f, 1735 cm^−1^ in F-W is the stretching vibration absorption peak of carbon base and carboxyl group C=O in integrated cellulose; 1637 cm^−1^ is the stretching vibration of conjugate alkene (contained in furfural). The characteristic absorption peaks of lignin are 1618, 1508 and 1460 cm^−1^. The vibration absorption peak of alcohols and phenols at 1247 cm^−1^ and the stretching vibration absorption peak of C–O in polysaccharides at 1060 cm^−1^. The split seam of the C-O stretching vibration absorption peak of the polysaccharide fission peak is 1166 cm^−1^. These peaks weakened or disappeared to different degrees, indicating that during the hot-pressing process, lignin, cellulose and hemicellulose were hydrolyzed and then turned into small molecules to undergo polymerization reaction and generate new polymers. In F-CS-V, 1637 cm^−1^ is absorption peak of conjugated alkene stretching vibration and C=N stretching vibration are superimposed. Meanwhile, this strength is weakened, which may be because the addition of CS-V further aggravates the polymerization reaction in the hot-pressing process [25,26,27,28]. At the same time, a new peak appeared at 1595 cm^−1^ (amide Ⅱ) and formed a new amide bond, which is the reason why F-CS-V showed good physical and mechanical properties. The new peak at 1350 cm^−1^ may be the hydroxyl absorption peak from further acetylation of vanillin and chitosan to the formation of the intermediate hemiacetal.

**Figure 4:**
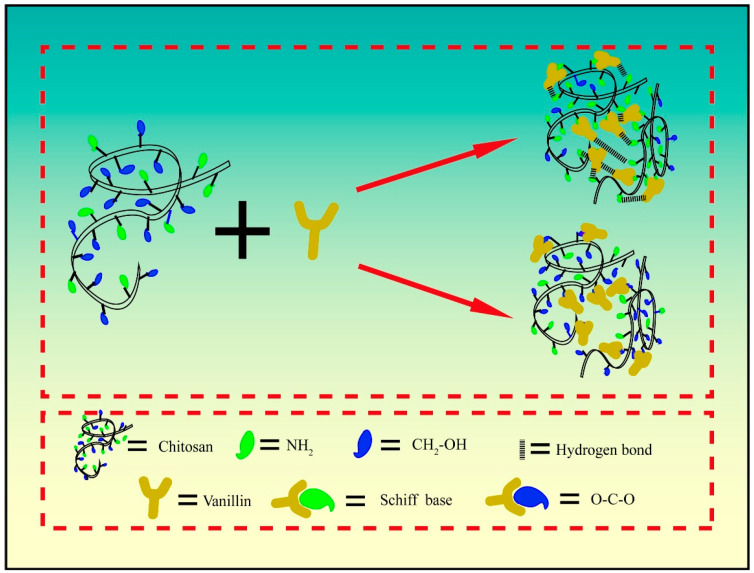
Cross-linking mechanism of vanillin and chitosan.

Figure 4 shows the cross-linking mechanism of vanillin crosslinked chitosan. On the one hand, before hot pressing, the amino group of CS reacts with the aldehyde group of V to form C=N and there is a hydrogen bond between the amino group and the phenol hydroxyl group. On the other hand, the CH_2_–OH of CS is acetal reaction with the aldehyde group of V after hot pressing.

### 3.4. NMR Analysis

**Figure 5:**
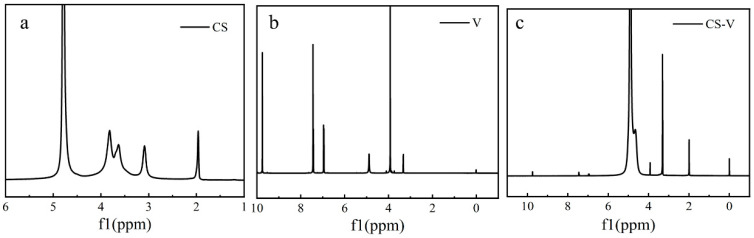
(**a**–**c**) is the ^1^HNMR spectra of CS, V and CS-V respectively.

Figure 5a–c present ^1^HNMR spectra of CS, V and CS-V respectively. In Figure 5a, the chemical shift at 4.79 ppm and 1.9 ppm are the solvent peak, the -OH peak at 3.6 ppm, and the C–H peak at 3.09 ppm. In Figure 5b, the chemical shift is the -CHO peak at 9.75 ppm and the phenol hydroxyl and benzene ring at 7.4 ppm and 6.9 ppm. The –O–CH_3_ peak is 3.93 ppm, and 3.3 ppm is the solvent peak. In Figure 5c, chemical shifts at 6.9 ppm and 5.0 ppm are the solvent peak. At the same time 3.4 ppm is –CH, and 2.0 ppm is -CH_2_. However, the chemical shifts at 9.75 ppm and 7.45 ppm emergence proton peak are the resonance peak of hydrogen atoms on the benzene ring. Combined with infrared analysis, Schiff base polymer is generated.

### 3.5. XRD Analysis

**Figure 6:**
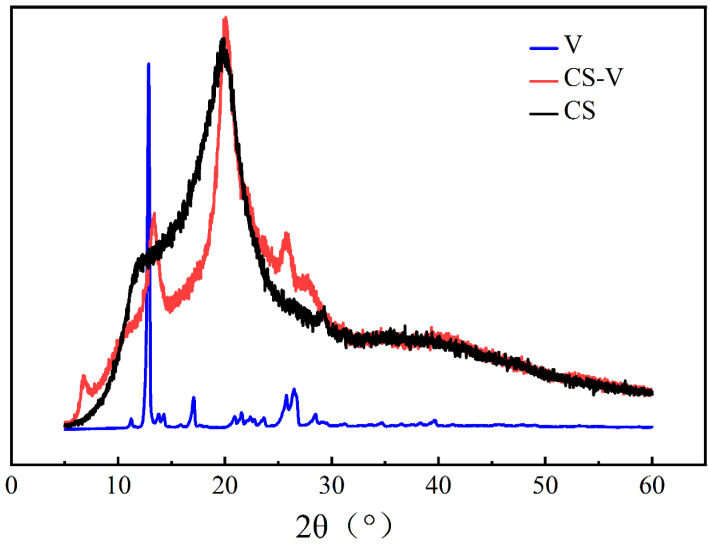
XRD patterns of CS, V and CS-V.

Figure 6 shows XRD pattern of CS, V and CS-V. Similar to the literature, diffraction peaks of CS are near 10° and 20° [27]. V has obvious diffraction peaks at 13° and 40° due to its crystalline properties [28]. Vanillin crosslinked chitosan has a new peak near 2θ = 7°, indicating that the crystallization characteristics of the crosslinked chitosan have changed significantly due to the emergence of a new combination bond in the process of vanillin and chitosan cross-linking curing [29,30]. Thus, reversible bonds in the hydrogel network contain two types of bonds, one of which is primarily the formation of Schiff base bonds (C=N) between the amino group in the CS molecule and the aldehyde group of V. The other is the 4-hydroxyl group of V with the amino or hydroxyl group of CS forming a physical hydrogen bond, which contributes to the formation of Schiff base bonds [20]. The diffraction peaks of crosslinked chitosan near 2θ = 13° and 27° are due to the incomplete reaction of V. 

### 3.6. TG Analysis

**Figure 7:**
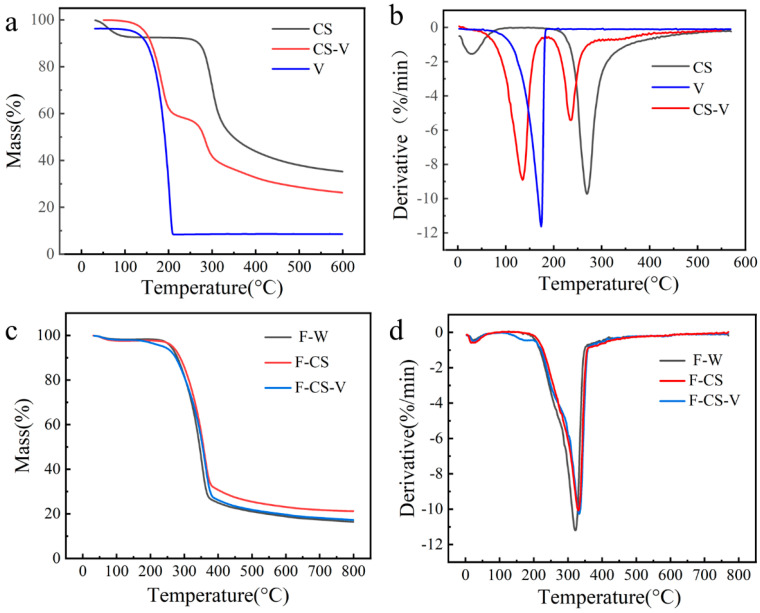
TG (**a**) and DTG (**b**) curve of CS, V and CS-V, TG (**c**) and DTG (**d**) curve of F-W, F-CS and F-CS-V.

Figure 7 shows the TG and DTG curves of CS, V, CS-V, F-W, F-CS and F-CS-V. In Figure 7a, the thermal decomposition of CS is divided into two stages, and the first stage between 30~96 °C, which is mainly water evaporation. The second order appears between 96~319 °C, which is mainly a solution to the dehydration and polymer unit of the sugar ring. The thermal decomposition phase of the CS-V was between 155~200 °C, which was mainly due to the excess of the vanilla, which was the largest at 184.9 °C. The decomposition stage of CS derivative was 270~298.6 °C. In Figure 7b, we can conclude that the thermal stability of CS-V is lower than that of CS. It is mainly due to the successful crosslinking of vanillin and chitosan that chitosan grafts new groups and breaks the hydrogen bond between chitosan molecules. Thus, its thermal stability decreases.

In Figure 7c, a comprehensive analysis of the weight loss data of F-W, F-CS and F-CS-V pyrolysis showed that the thermal decomposition of F-W, F-CS and F-CS-V can be divided into three stages and the maximum weight loss rate of the samples was achieved in the second stage, which was due to the degradation of cellulose, hemicellulose and lignin [31]. in Figure 7d, F-W at 320 °C, F-CS at 330 °C, F-CS-V at 337 °C DTG curves appear when maximum peak, F-CS-V peak temperature is higher than that of the other samples which suggests that crosslinked chitosan joined to a certain extent, can increase the thermal stability of the fiberboard.

### 3.7. XPS Analysis

**Figure 8:**
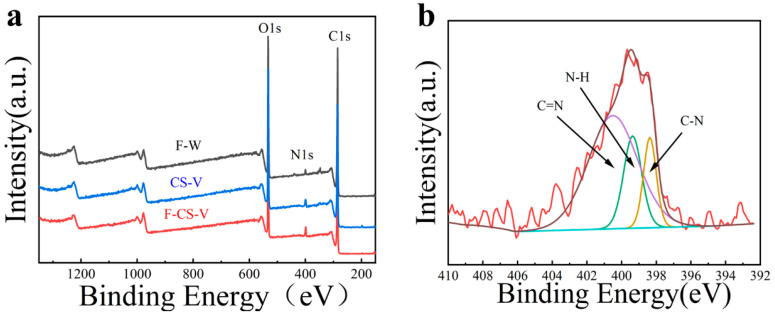
(**a**) XPS spectra of F-W, CS-V and F-CS-V, (**b**) N1s spectra of F-CS-V.

Figure 8 shows the XPS spectra of F-W, CS-V and F-CS-V. As for the Figure 8a above, black curve (F-W), blue curve (CS-V) and red curve (F-CS-V) have two main characteristic peaks at 285.1 eV and 533.1 eV, which are attributed to C1 and O1s, respectively [32,33]. Compared with F-W, F-CS-V had a more obvious characteristic peak at 399.1 eV, corresponding to N1s, indicating that the surface of wood fiber was successfully covered by nitrogen-containing crosslinked CS. As can be seen from the N1s spectra of F-CS-V (Figure 7b), fitting peak at 398.8 eV is N-C groups, fitting peak at 399.3 eV is N-H groups, fitting peak at 400.5 eV is N=C groups. The amino group of CS formed N=C groups through a reaction with the aldehyde group of V. The results showed that V reacted with CS to create a Schiff base.

## 4. Conclusions

In conclusion, considering the problem of formaldehyde release in traditional MDF, crosslinking agents with low toxicity are often used in chitosan-based binders. Natural environmental protection vanillin was used as crosslinking agent. At the same time crosslinked chitosan was used as adhesive, when the ratio of vanillin to chitosan was 2:1, MDF with good physical and mechanical properties of MOR (20.64 MPa), MOE (3005 MPa), IB (0.86 MPa) and TS (14.7%) was successfully prepared. These results are superior to those of wood fiber composites with chitosan as binder. The reason for this is that the newly formed amide bond and hydrogen bond greatly improve the mechanical properties. This method provides reference and idea for preparing environmental protection fiberboard.

## Figures and Tables

**Table 1:** Stoichiometry of chemical reagents for the preparation of crosslinked chitosan.

Mass Ratio ^a^	Chitosan (g)	Acetic Acid (g)	Vanillin (g)	Deionized Water (mL)
0.5:1	3.24	3.5	1.62	270
1:1	3.24	3.5	3.24	270
1.5:1	3.24	3.5	4.86	270
2:1	3.24	3.5	6.48	270

^a^ The mass ratio refers to vanillin to chitosan.

## Data Availability

Not applicable.
